# Respiratory infections in X-linked hyper-IgM syndrome with *CD40LG* mutation: a case series of seven children in China

**DOI:** 10.1186/s12887-022-03726-z

**Published:** 2022-11-22

**Authors:** Huifeng Fan, Li Huang, Diyuan Yang, Changhao Zhang, Qiang Zeng, Genquan Yin, Gen Lu, Kunling Shen

**Affiliations:** 1 Department of Respiratory Medicine, China National Clinical Research Center of Respiratory Diseases, Beijing Children’s Hospital, Capital Medical University, National Center for Children’s Health, Beijing, 100045 China; 2 Department of Respiration, Guangzhou Women and Children’s Medical Center, Guangzhou Medical University, Guangzhou, 510120 China; 3 Pediatric Intensive Care Unit, Guangzhou Women and Children’s Medical Center, Guangzhou Medical University, Guangzhou, 510120 China

**Keywords:** X-linked hyper-immunoglobulin M syndrome, Respiratory infection, Children, Pathogen, Mutation

## Abstract

**Background:**

X-linked hyper-immunoglobulin M (XHIGM), a primary immunodeficiency syndrome caused by mutations in the CD40 ligand gene(*CD40LG*), presents with recurrent respiratory infections in pediatric patients. We aimed to evaluate the spectrum of clinical features and respiratory pathogens in pediatric patients with XHIGM in China.

**Methods:**

We retrospectively reviewed seven pediatric patients who were diagnosed with XHIGM and received follow-up treatment at the Guangzhou Women and Children’s Medical Center between January 2010 and January 2021. We determined their clinical characteristics, causative pathogens, and prognosis by performing peripheral immunological and genetic tests.

**Results:**

There were seven boys with age ranging from 4–20 months (median age, 13 months). Four of the seven respiratory infections were caused by *Talaromyces marneffei(T. marneffei)*. Two patients had viral infections caused by cytomegalovirus (CMV) and human adenovirus respectively. One patient had a mixed infection caused by *Pneumocystis carinii* and CMV. Except for one child who died of respiratory failure, one patient received hematopoietic stem cell transplantation (HSCT) and recovered well, the other five patients survived with regular infusions of intravenous immunoglobulin (IVIg) during the follow-up period. Six patients had reduced antibody levels, especially IgG, IgA, and IgE levels. Increased serum IgM levels were detected in four cases, and three cases presented normal IgM levels at onset. All children were diagnosed with XHIGM with *CD40LG* variation. Three novel mutations were identified in the present study.

**Conclusions:**

Our study suggests that respiratory infections usually begin within 2 years old, fungi and viruses are important pathogens causing respiratory infections in children with XHIGM. In endemic areas, *T. marneffei* is the common pathogen of respiratory tract infection in children with the disease.

**Supplementary Information:**

The online version contains supplementary material available at 10.1186/s12887-022-03726-z.

## Introduction

Hyper-immunoglobulin M syndrome (HIGM) is a rare primary immunodeficiency, in which defective B cell isotype switching leads to a phenotype characterized by low or absent serum levels of IgG, IgA and IgE, whereas the IgM concentration is either normal or increased [1–4]. This syndrome is known as immunoglobulin class switch recombination (Ig-CSR) deficiencies. Mutations in a number of genes involved in CSR have been implicated in the development of HIGM profile, including CD40 ligand (CD40L), CD40, nuclear factor kappa-B essential modulator (NEMO/IKKγ), inhibitor of kappa light-chain gene enhancer in B cells, alpha (IκBα), nuclear factor kappa-B subunit 1 (NFKB1), activation-induced cytidine deaminase (AICDA/AID), and so on [2, 5, 6]. The X-linked Hyper-IgM syndrome (XHIGM), which is caused by CD40L mutations, is the most common form of HIGM and accounts for about 65–70% of all cases [7, 8]. The prevalance of XHIGM was approximately 1 in 1,000,000 live births. CD40L is expressed primarily on activated CD4^+^ T cells, and interacts with CD40 expressed on B cells, monocytes, macrophages, and dendritic cells [3]. Therefore, XHIGM is confirmed as the combined T and B immunological deficiency, and it is clearly illustrated by the susceptibility of patients with XHIGM to recurrent pyogenic and opportunistic infections [9].

Clinical manifestations of XHIGM are complex and severe. Patients with XHIGM due to the defective interaction between T and B. Therefore, they are more susceptible to infections that depend on IgA, IgG and IgE, with repetitive episodes of sinusitis, otitis, tonsillitis, cutaneous infections, giardiasis, helminthiasis, enterovirus meningitis, pneumonia, in addition to impairment of weight and height gain, and a tendency to autoimmune diseases and neoplasms [10]. The majority of patients with XHIGM develop recurrent pulmonary infections in the first 2 years of life [11]. Some patients present to medical attention with *Pneumocystis carinii* (*P.carinii*) pneumonia during their first year of life [11, 12]. This retrospective study on seven children diagnosed with XHIGM preliminarily assessed the clinical presentation and unusual pathogens of respiratory infections in these patients and aimed to describe the respiratory infection characteristics of these patients.

## Methods

### Patients and ethics

The data of seven patients with XHIGM admitted to Guangzhou Women and Children’s Medical Center between January 2010 and January 2021 were retrospectively analyzed. Clinical data including demographic characteristics (age and gender), clinical symptoms and signs, laboratory data, microbiological findings, imaging examinations, complications, treatments, and outcomes were collected and analyzed.

The study was conducted in accordance with the Declaration of Helsinki, and the protocol was approved by the Ethics Committee of Guangzhou Women and Children’s Medical Center of Guangzhou Medical University (Grant No. 2021084A01). Written informed consent was obtained from the children’s parents or legal guardians for the use of their clinical and laboratory data from their medical reports.

### Diagnostic methods

Patients were diagnosed with XHIGM based on the clinical characteristics and genetic test results of the patients. The updated classification of primary immunodeficiency disorders (PIDs) was performed to the standards as diagnostic criteria by the Primary Immunodeficiency Expert Committee of the International Union of Immunological Societies [13]. Selective PID panel testing (MyGenostics, Inc., China), a molecular diagnosis system based on targeted region sequencing of candidate genes, was performed on our patients.

Nasopharyngeal swab and sputum, bronchoalveolar lavage fluid (BALF), pleural fluid, and/or serum samples were collected for tests including immunofluorescence staining, antigen and antibody tests, nucleic acid detection, and bacterial culture. Pathogen high-throughput genome sequencing (KingMed Diagnostics, Inc., China) of BALF was performed in four patients to screen for bacteria, viruses, fungi, parasites, *Mycobacterium tuberculosis* complex, *Mycoplasma*, and *Chlamydia*.

## Results

### Clinical characteristics and pathogens

**Fig. 1:**
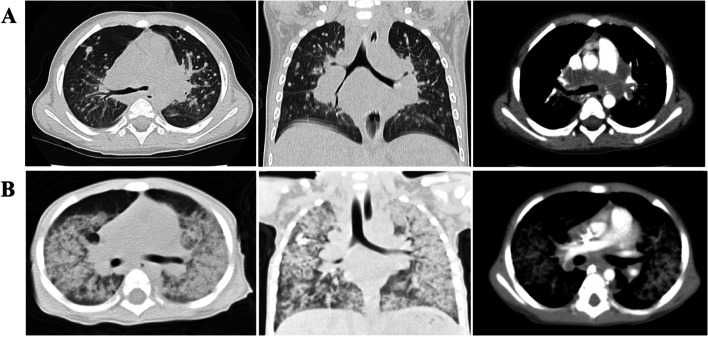
Lung windows from high-resolution CT scan of the patients. **A** Diffused nodules on two lungs with enlarged hilar lymphatic nodules (white arrows) in P1; **B** Bilateral distributing ground-glass opacities in P3

The clinical features of the children are summarized in Table 1. There were seven boys with age ranging from 4–20 months (median age, 13 months). All children had respiratory infections, including fever and cough. All patients had at least one episode of respiratory tract infection before diagnosis at first hospitalization in our center, four (P1, P5, P6, and P7) had *Talaromyces marneffei(T. marneffei)* infection and two (P2 and P3) were identified to have cytomegalovirus (CMV) and human adenovirus (HAdV) infections, respectively; Only P2 was confirmed to have a mixed infection caused by *P. carinii* and CMV. From a pulmonary imaging perspective, four patients developed lymphadenopathy and diffuse infiltration with *T. marneffei* infection on high-resolution computed tomography (HRCT) (Fig. 1A). The other three patients (P2, P3, and P4) showed ground-glass opacities on HRCT (Fig. 1B). Specifically, seven patients were followed up for 3–10 years. During the follow-up period, only P3 died of respiratory failure.P4 undertook hematopoietic stem cell transplantation (HSCT), and quite a good recovery of hemopoiesis was observed 6 months after HSCT. In addition, the others survived with regular infusions of intravenous immunoglobulin (IVIg).

**Table 1:** Clinical features of respiratory infection in the children with XHIGM

Patients	Age (months)	Gender	Respiratory symptoms and signs	Pathogens	Pulmonary imaging	Prognosis
1	13	M	Cough, fever, polypnea	*T. marneffei* (BALF,Airway mucosal biopsy)	Multiple patchy, Hilar lymphadenopathy	Survival
2	6	M	Cough, fever, polypnea	*P. carinii*, CMV(BALF)	Ground-glass opacity	Survival
3	4	M	Cough, fever, polypnea	HAdV(BALF, Nasopharyngeal swabs)	Ground-glass opacity	Died
4	5	M	Cough, polypnea	CMV(Sputum)	Ground-glass opacity	Survival
5	19	M	Cough, fever	*T. marneffei*(Blood)	Multiple nodules, Hilar and mediastinal lymphadenopathy	Survival
6	15	M	Cough, fever, wheeze, polypnea	*T. marneffei*(Blood)	Hydrothorax, Hilar and mediastinal lymphadenopathy	Survival
7	20	M	Cough, fever	*T. marneffei*(Airway mucosal biopsy)	Multiple nodules and patchy, Hilar lymphadenopathy	Survival

*T marneffei* Talaromyces marneffei, *P carinii* Pneumocystis carinii*, BALF* Bronchoalveolar lavage fluid, *HAdV* Human adenovirus, *CMV* Cytomegalovirus

### Hematological and immunological parameters

Immunological evaluations of the seven patients at the time of diagnosis are listed in Table 2 and Table S1. Almost all patients had reduced serum IgG levels, except for one child (P6) treated with IVIg before the examination. Serum IgM levels of P3, P4, and P5 were slightly elevated, while serum IgM levels of the other patients (P1, P2, P6, and P7) were normal. High or normal T and B lymphocyte counts were observed in all patients. The counts of CD19^+^ B cells were elevated in P1, P2, P3, P4, and P7, whereas the other two patients (P5 and P6) had normal CD19^+^ B cell counts. Nitro blue tetrazolium test results were normal in all patients. Four children (P1, P4, P5 and P7) were found temporary neutropenia, the ratio of neutrophils decreased in most patients (P1, P2, P3, P4 and P7).

**Table 2:** Immunoassays findings of the children with XHIGM^a^

Patients	IgG(g/L) (Normal range)	IgA(g/L) (Normal range)	IgM(g/L) (Normal range)	IgE(IU/ML) (Normal range)	CD19 + Abs(cells/ul) (Normal range)	CD3 + 4 + (cells/ul) (Normal range)	CD3 + 8 + (cells/ul) (Normal range)	CD56 + (cells/ul) (Normal range)	NBT test
1	0.52 (3.52–10.69)	0.1 (0.13–1.38)	0.53 (0.38–2.03)	< 5 (0–60)	2279.28 (90–660)	1746.12 (410–1590)	431.18 (190–1140)	161 (90–590)	Normal
2	0.33 (3.6–9.2)	0.07 (0.08–0.56)	0.48 (0.38–1.26)	< 5 (0–60)	3440.47 (240–1317)	3470.7 (345–2350)	1628.6 (314–2080)	561.13 (210–1514)	Normal
3	0.88–1.26 (3.06–7.74)	0.07 (0.07–0.41)	2.63–5.45 (0.24–0.88)	< 5 (0–15)	2295.16 (240–1317)	6745.74 (345–2350)	2372.63 (314–2080)	217.89 (210–1514)	Normal
4	0.71 (3.06–7.74)	0.07 (0.07–0.41)	1.34 (0.24–0.88)	< 5 (0–15)	3441.95 (240–1317)	2365.98 (345–2350)	1824.7 (314–2080)	234.14 (210–1514)	Normal
5	< 0.33 (3.82–10.58)	0.17 (0.14–1.14)	2.65 (0.4–1.28)	< 5 (0–60)	364.43 (90–660)	1458.66 (410–1590)	347.75 (190–1140)	262.96 (90–590)	Normal
6	9.79^b^ (3.82–10.58)	0.07 (0.14–1.14)	0.58 (0.4–1.28)	< 5 (0–60)	395.23 (90–660)	1973.05 (410–1590)	1706.35 (190–1140)	247.34 (90–590)	Normal
7	1.49 (3.82–10.58)	0.07 (0.14–1.14)	0.48 (0.4–1.28)	< 5 (0–60)	1945.34 (90–660)	3323.59 (410–1590)	1350.24 (190–1140)	479.35 (90–590)	Normal

^a^The normal ranges were presented because they changed with different kit and different age

^b^The tests were conducted after IVIg treatment

*NBT* Nitro blue tetrazolium test

### Mutation studies

Using selective PID panel testing, all patients were found to have mutations in the *CD40LG* gene (Table 3) and were diagnosed with XHIGM. In this cohort, hemizygous mutations were found in five patients (P1, P2, P3, P4, and P7), and microdeletions were found in two others (P5 and P6). The mutations were mostly inherited via the maternal lineage (P1, P2, P5, P6, and P7). However, parents of P3 and P4 did not carry the same mutation as their children. Two novel hemizygous mutations (c.424_436del and c.488delT) in exon 5 were detected in P1 and P3, respectively, whereas a novel hemizygous mutation (c.92_104del) in exon 1 was found in P2. These mutations have never been reported in gnomAD and were predicted to be disease-causing mutations using PolyPhen2, Mutation Taster, PROVEAN, and SIFT software.

**Table 3:** Genetic detection of the children with XHIGM

Patient	Gene	Genetic variation	Source	Nucleotide variation	Amino acid mutation	Amino acid variation
1	*CD40LG*	Hemizygote	Mother	c.424_436del	Frameshift mutation	p.E142Tfs*3
2	*CD40LG*	Hemizygote	Mother	c.92_104del	Frameshift mutation	p.F31Lfs
3	*CD40LG*	Hemizygote	Spontaneous mutation	c.488del	Frameshift mutation	p.R165Dfs*26
4	*CD40LG*	Hemizygote	Spontaneous mutation	c.761C > T	Missense mutation	p.T254M
5	*CD40LG*	Microdeletion	Mother	> 132 kb	-	-
6	*CD40LG*	Microdeletion	Mother	c.1978 + 1G > A	-	-
7	*CD40LG*	Hemizygote	Mother	c.598A > T	Nonsense mutation	p.R200*

## Discussion

XHIGM is a primary combined immunodeficiency syndrome that results from mutations in the *CD40LG*, which encodes for the CD40L protein [2]. This retrospective analysis of seven pediatric patients aimed to collect data on the clinical presentation, treatment, and causative respiratory pathogens of patients with XHIGM and use this information to gain further understanding of clinical features and the pathogens of respiratory infections in pediatric patients with XHIGM.

The most prominent clinical feature observed in XHIGM patients is recurrent respiratory infection, the symptoms usually develop in infancy and second year of life [3]. In our study, all seven boys suffered respiratory infections by the age of 2, and they had at least one episode of respiratory tract infection before a diagnosis is made. Furthermore, XHIGM results in combined immune deficiency with a defect in Ig isotype switching and enhanced susceptibility to opportunistic infections [14]. According to the European and US cohorts, the most common pathogen causing opportunistic infections is *P. carinii,* while others include *Bartonella*, *Cryptosporidium parvum*, *Histoplasma*, *Mycobacterium avium*, *Salmonella dublin*, *Herpes* family viruses*,* etc. [3, 14, 15]. In this study, only P2 had *P. carinii* infection, as previously reported, while four patients (P1, P5, P6, and P7) were found to have *T. marneffei* infection in the lungs. *T. marneffei* is an opportunistic pathogen that infects immunodeficient patients in Southeast Asia as a dimorphic fungus, and *T. marneffei* infection is a severe disease that can lead to high mortality rates of greater than 50% in children in previous reports [16]. In adults, *T. marneffei* infection has been considered to be exclusively associated with acquired immunodeficiency syndrome (AIDS) caused by human immunodeficiency virus (HIV) infection [17]. In contrast to adults, pediatric patients with PIDs are more susceptible to *T. marneffei* according to previous reports [16, 18]. Therefore, pediatricians should be alert to *T. marneffei* respiratory infection in XHIGM patients in high-prevalence areas.

It should be noted that viral infections have also been linked to the development of respiratory infections in these children [3, 12]. In our study, three patients presented with interstitial pneumonia with extensive ground-glass opacity on HRCT, due to *P. carinii* and other viral infections. Of these, a unique case of HAdV infection (P3) progressively worsened and the patient eventually died of respiratory failure. What needs to pay attention is that all three patients began with the respiratory symptoms within 6 months of birth as first infection. Of note, interstitial lung disease in infancy should be paid more attention to XHIGM by pediatric clinicians even for the first time.

XHIGM with *CD40LG* mutations has been classified as combined T and B immunodeficiency, and the overall prognosis of these patients is poor, with an overall survival rate of 20–28.2% [9, 14, 19, 20]. Earlier management with IVIg replacement therapy and antibiotic prophylaxis could improve the quality of life of patients by reducing the incidence of life-threatening infections [3]. In this study, most confirmed cases were regularly administered IVIg replacement therapy, and no serious infections occurred in them during the follow-up years. Hematopoietic stem cell transplantation (HSCT) represents the only curative choice available for patients with XHIGM [21]. However, the presence of severe pre-transplant infections, organ damage (usually affecting the liver and/or lung), lack of a matched donor, or socioeconomic constraints, while age at transplantation seems to be the major challenge. Recent research found a more favorable outcome and greater event-free survival among patients who underwent transplantation before age 6 years in comparison with patients 6 years or older [22]. In our cohort, only one patient has been performed HSCT and recovered well, while the other patients had not been treated with HSCT for the matched donor's lack or economic reason. IVIg replacement therapy is still the first choice for XHIGM patients to prevent infection before HSCT.

XHIGM is a PID caused by mutations in the *CD40LG* gene. *CD40LG* is located on the X chromosome, which encodes CD40L (CD154), a member of the tumor necrosis factor family made-up of three functional domains: intracellular, transmembrane and extracellular domains [23]. Since CD40L is necessary for T lymphocytes to induce B lymphocytes to undergo class switching from IgM to IgG, IgA, and IgE, patients with XHIGM syndrome have reduced levels of IgG, IgA, and IgE, and normal or elevated levels of IgM [8, 9]. In this study, our patients had typical immune parameters of XHIGM at onset, which indicated the possibility of XHIGM and the necessity for genetic testing. As expected, mutations in *CD40LG* were found in all seven pediatrics patients. Two patients were diagnosed with XHIGM due to microdeletions of the *CD40LG* gene (P5 and P6). Interestingly, three cases of novel hemizygote mutations have not been reported in gnomAD so far (P1, P2, and P3), where other variants at the same position are found. Based on the clinical manifestations and pedigree analysis of the patients according to the American College of Medical Genetics variation classification guidelines [24], the three mutations can be taken as evidence for pathogenicity. Future research could focus on functional verification by animal experimentation.

### Strengths

This study emphasized fungi and viruses are important pathogens of respiratory infections in pediatric patients with XHIGM. In our cohort, patients were more susceptible to *T. marneffei*, which suggested *T. marneffei* infections could be a clinical clue to realize XHIGM for pediatricians. For infant, interstitial lung disease could be the first symptom of respiratory infection in XHIGM patients. In addition, three novel mutations in *CD40LG* were first reported in our cohort.

### Limitations

This study has some limitations that should be noted. This was a single-center study with a limited number of cases. CD40 ligand expression levels in in vitro-activated lymphocytes should be assayed for the patients, but it has not yet been carried out in our hospital, it is great regret that our patients failed to get tested. Nevertheless, this study provides a valuable reference for respiratory infections, especially fungal and viral infections, in children with XHIGM.

## Conclusions

XHIGM patients usually presents respiratory infections within 2 years old, and fungi and viruses are important respiratory pathogens. *T. marneffei* respiratory infections were more common in children with XHIGM in endemic areas. Infants who were diagnosed with interstitial lung disease should be alert to XHIGM. In this study, we presented three novel mutations in *CD40LG* that have not been reported previously.

## Supplementary Information

**Additional file 1:** **Table S1.** Blood routine test findings of the children with XHIGM.

## Data Availability

The datasets generated and/or analyzed during the current study are not publicly available due individual privacy of patients could be compromised, but are available from the corresponding author on reasonable request.

## Abbreviations

XHIGM: X-linked hyper-immunoglobulin M
*T*. marneffei: Talaromyces marneffei
CMV: Cytomegalovirus
IVIg: Intravenous immunoglobulin
PID: Primary immunodeficiency
HIGM: Hyper-immunoglobulin M
Ig-CSR: Immunoglobulin class-switch recombination
CD40L: CD40 ligand
BALF: Bronchoalveolar lavage fluid
HAdV: Human adenovirus
*P.* carinii: Pneumocystis carinii
HRCT: High-resolution computed tomography
AIDS: Acquired immunodeficiency syndrome
HSCT: Hematopoietic stem cell transplantation

## References

[1] Tafakori Delbari M, Cheraghi T, Yazdani R, Fekrvand S, Delavari S, Azizi G (2019). Clinical Manifestations, Immunological Characteristics and Genetic Analysis of Patients with Hyper-Immunoglobulin M Syndrome in Iran. Int Arch Allergy Immunol.

[2] Moazzami B, Yazdani R, Azizi G, Kiaei F, Tafakori M, Modaresi M (2019). Respiratory Complications in Patients with Hyper IgM Syndrome. J Clin Immunol.

[3] Yazdani R, Fekrvand S, Shahkarami S, Azizi G, Moazzami B, Abolhassani H (2019). The hyper IgM syndromes: Epidemiology, pathogenesis, clinical manifestations, diagnosis and management. Clin Immunol.

[4] Meng X, Yang B, Suen WC (2018). Prospects for modulating the CD40/CD40L pathway in the therapy of the hyper-IgM syndrome. Innate Immun.

[5] Aghamohammadi A, Parvaneh N, Rezaei N, Moazzami K, Kashef S, Abolhassani H (2009). Clinical and laboratory findings in hyper-IgM syndrome with novel CD40L and AICDA mutations. J Clin Immunol.

[6] Picard C, Bobby Gaspar H, Al-Herz W, Bousfiha A, Casanova JL, Chatila T (2018). International Union of Immunological Societies: 2017 Primary Immunodeficiency Diseases Committee Report on Inborn Errors of Immunity. J Clin Immunol.

[7] Matamoros Flori N, Mila Llambi J, Espanol Boren T, Raga Borja S, Fontan CG (1997). Primary immunodeficiency syndrome in Spain: first report of the National Registry in Children and Adults. J Clin Immunol.

[8] Tsai HY, Yu HH, Chien YH, Chu KH, Lau YL, Lee JH (2015). X-linked hyper-IgM syndrome with CD40LG mutation: two case reports and literature review in Taiwanese patients. J Microbiol Immunol Infect.

[9] Winkelstein JA, Marino MC, Ochs H, Fuleihan R, Scholl PR, Geha R (2003). The X-linked hyper-IgM syndrome: clinical and immunologic features of 79 patients. Med (Baltimore).

[10] Leite LFB, Maximo TA, Mosca T, Forte WCN (2020). CD40 Ligand Deficiency. Allergol Immunopathol (Madr).

[11] Qamar N, Fuleihan RL (2014). The hyper IgM syndromes. Clin Rev Allergy Immunol.

[12] Levy J, Espanol-Boren T, Thomas C, Fischer A, Tovo P, Bordigoni P (1997). Clinical spectrum of X-linked hyper-IgM syndrome. J Pediatr.

[13] Picard C, Al-Herz W, Bousfiha A, Casanova JL, Chatila T, Conley ME (2015). Primary Immunodeficiency Diseases: an Update on the Classification from the International Union of Immunological Societies Expert Committee for Primary Immunodeficiency 2015. J Clin Immunol.

[14] Madkaikar M, Gupta M, Chavan S, Italia K, Desai M, Merchant R (2014). X-linked hyper IgM syndrome: clinical, immunological and molecular features in patients from India. Blood Cells Mol Dis.

[15] Ochs HD (2008). Patients with abnormal IgM levels: assessment, clinical interpretation, and treatment. Ann Allergy Asthma Immunol.

[16] Guo J, Li BK, Li TM, Wei FL, Fu YJ, Zheng YQ (2019). Characteristics and Prognosis of Talaromyces marneffei Infection in Non-HIV-Infected Children in Southern China. Mycopathologia.

[17] Limper AH, Adenis A, Le T, Harrison TS (2017). Fungal infections in HIV/AIDS. Lancet Infect Dis.

[18] Fan H, Huang L, Jin Y, Chen C, Lu G, Zhang D (2017). Study ofPenicillium marneffeiInfection in Pediatric Patients Without Human Immunodeficiency Virus Infection in China. Pediatr Allergy Immunol Pulmonol.

[19] Cabral-Marques O, Klaver S, Schimke LF, Ascendino EH, Khan TA, Pereira PV (2014). First report of the Hyper-IgM syndrome Registry of the Latin American Society for Immunodeficiencies: novel mutations, unique infections, and outcomes. J Clin Immunol.

[20] de la Morena MT, Leonard D, Torgerson TR, Cabral-Marques O, Slatter M, Aghamohammadi A (2017). Long-term outcomes of 176 patients with X-linked hyper-IgM syndrome treated with or without hematopoietic cell transplantation. J Allergy Clin Immunol.

[21] Castagnoli R, Delmonte OM, Calzoni E, Notarangelo LD (2019). Hematopoietic Stem Cell Transplantation in Primary Immunodeficiency Diseases: Current Status and Future Perspectives. Front Pediatr.

[22] Franca TT, Barreiros LA, Salgado RC, Napoleao S, Gomes LN, Ferreira JFS (2022). CD40 Ligand Deficiency in Latin America: Clinical, Immunological, and Genetic Characteristics. J Clin Immunol.

[23] Karpusas M, Hsu YM, Wang JH, Thompson J, Lederman S, Chess L (1995). 2 A crystal structure of an extracellular fragment of human CD40 ligand. Structure.

[24] Riggs ER, Andersen EF, Cherry AM, Kantarci S, Kearney H, Patel A (2020). Technical standards for the interpretation and reporting of constitutional copy-number variants: a joint consensus recommendation of the American College of Medical Genetics and Genomics (ACMG) and the Clinical Genome Resource (ClinGen). Genet Med.

