# Supplementary material for: Respiratory infections in X-linked hyper-IgM syndrome with CD40LG mutation: a case series of seven children in China
Source: BMC Pediatr. 2022 Nov 22;22:675. doi: 10.1186/s12887-022-03726-z (PMC9682706; doi:10.1186/s12887-022-03726-z)
Supplement: Supplementary file 1 — Additional file 1: Table S1. Blood routine test findings of the children with XHIGM. [file 12887_2022_3726_MOESM1_ESM.docx]

| **Table S1** Blood routine test findings of the children with XHIGM | | | | | | |
| --- | --- | --- | --- | --- | --- | --- |
| **Patients** | **Leukocyte count (×10^9^/L)**  **(5-12)** | **Neutrophil count (×10^9^/L)**  **(2.0-7.2)** | **Neutrophil ratio**  **(%)**  **(31-40)** | **Lymphocyte count (×10^9^/L)**  **(1.55-4.8)** | **Hemoglobin**  **(g/dL) (105-145)** | **Platelet count**  **(×10^12^/L)**  **(140-440)** |
| 1 | 3.7-15.7 | 0.67-6.28 | 19-80 | 0.9-7.68 | 60-146 | 23-465 |
| 2 | 10.9-23.4 | 2.81-12.17 | 16-52 | 6.49-13.32 | 98-123 | 519-871 |
| 3 | 17.5-32.6 | 2.25-11.5 | 13-49 | 8.83-19.23 | 102-126 | 384-819 |
| 4 | 13.6-21.3 | 1.26-7.07 | 8-33 | 9.19-17.50 | 105-120 | 443-627 |
| 5 | 2.2-13.9 | 1.22-8.02 | 38-55 | 0.81-5.11 | 43-143 | 21-220 |
| 6 | 20.5 | 13.73 | 67 | 5.13 | 102 | 173 |
| 7 | 2.8-16.1 | 0.1-5.35 | 3-41 | 1.22-8.38 | 87-115 | 221-852 |
| The results were collected during their hospitalizations. | | | | | | |
